# A Cytokine-Related Gene Signature for Pan-Cancer Prognostic Stratification and Malignant Phenotype Characterization

**DOI:** 10.3390/ijms27062830

**Published:** 2026-03-20

**Authors:** Shih-Chieh Chen, Kai-Fu Chang, Chien-Cheng Chao, Chung-Hsien Lin, Chih-Hsuan Chang, Ching-Chung Ko, Hui-Ru Lin, Chi-Jen Wu, Chien-Han Yuan, Sachin Kumar, Dahlak Daniel Solomon, Do Thi Minh Xuan, Neethu Palekkode, Ayman Fathima, Junanda Waikhom, Chih-Yang Wang, Yung-Kuo Lee, Hui-Pu Liu

**Affiliations:** 1 Department of Internal Medicine, Kaohsiung Armed Forces General Hospital, National Defense Medical University, Kaohsiung 80284, Taiwan; eschatologyorcus@hotmail.com; 2 Medical Laboratory, Medical Education and Research Center, Kaohsiung Armed Forces General Hospital, National Defense Medical University, Kaohsiung 80284, Taiwan; zjack5109@gmail.com (K.-F.C.); g1118020022@mail.802.org.tw (C.-C.C.); joey2719@gmail.com (C.-H.L.); abstyle0204@gmail.com (C.-H.C.); han86449@gmail.com (C.-H.Y.); yungkuolee@gmail.com (Y.-K.L.); 3 Division of Experimental Surgery Center, Department of Surgery, Tri-Service General Hospital, National Defense Medical University, Taipei 11490, Taiwan; 4 Department of Medical Imaging, Chi-Mei Medical Center, Tainan 71004, Taiwan; kocc0729@gmail.com; 5 Department of Health and Nutrition, Chia Nan University of Pharmacy and Science, Tainan 71710, Taiwan; 6 School of Medicine, College of Medicine, National Sun Yat-Sen University, Kaohsiung 80424, Taiwan; 7 Institute of Medical Science and Technology, National Sun Yat-Sen University, Kaohsiung 80424, Taiwan; linlulu0805@gmail.com; 8 Nursing Department, Kaohsiung Armed Forces General Hospital, National Defense Medical University, Kaohsiung 80284, Taiwan; iris189212@gmail.com; 9 College of Nursing, Kaohsiung Medical University, Kaohsiung 80708, Taiwan; 10 Department of Otolaryngology, Kaohsiung Armed Forces General Hospital, National Defense Medical University, Kaohsiung 80284, Taiwan; 11 Department of Otolaryngology, National Defense Medical University, Taipei 11490, Taiwan; 12 PhD Program for Cancer Molecular Biology and Drug Discovery, College of Medical Science and Technology, Taipei Medical University, Taipei 11031, Taiwan; sachinkumar1@shooliniuniversity.com (S.K.); dahlak1daniel@gmail.com (D.D.S.); pneethu2001@gmail.com (N.P.); jwaikhom1@gmail.com (J.W.); chihyang@tmu.edu.tw (C.-Y.W.); 13 Graduate Institute of Cancer Biology and Drug Discovery, College of Medical Science and Technology, Taipei Medical University, Taipei 11031, Taiwan; fathimaayman595@gmail.com; 14 Faculty of Applied Sciences and Biotechnology, Shoolini University of Biotechnology and Management Sciences, Solan 173229, India; 15 Yogananda School of AI Computers and Data Sciences, Shoolini University, Solan 173229, India; 16 Faculty of Pharmacy, Van Lang University, 69/68 Dang Thuy Tram Street, Binh Loi Trung Ward, Ho Chi Minh City 70000, Vietnam; xuan.dtm@vlu.edu.vn; 17 Department of Biotechnology, Mother Teresa Women’s University, Kodaikanal 624101, India; 18 Computer Engineering with Specialization in Artificial Intelligence and Machine Learning, Presidency University, Yelahanka, Bengaluru 560064, India; 19 Department of General Surgery, Kaohsiung Armed Forces General Hospital, National Defense Medical University, Kaohsiung 80284, Taiwan

**Keywords:** cytokines, pan-cancer, prognostic signature, tumor microenvironment, survival analysis, inflammation

## Abstract

Cytokines are central regulators of inflammation and immune responses within the tumor microenvironment and have been implicated in cancer progression and prognosis. However, the prognostic value of coordinated cytokine-related transcriptional programs across cancer types has not been systematically explored. Pan-cancer transcriptomic and clinical data were analyzed to construct a cytokine-related prognostic signature using least absolute shrinkage and selection operator (LASSO) Cox regression. Patients were stratified into high-risk and low-risk groups based on the derived risk score. Prognostic performance was evaluated in training and test cohorts, and biological relevance was assessed through survival analyses and pathway-level investigations. A 16-gene cytokine-related signature was established that consistently stratified patients into distinct prognostic groups across multiple cancer types. High cytokine-related risk scores were significantly associated with unfavorable survival outcomes and were linked to enhanced cell cycle activity, epithelial-mesenchymal transition, and extracellular matrix remodeling. Integration of the risk score with clinical variables improved individualized survival prediction. Immunohistochemical analyses further confirmed increased protein expression of representative risk-associated genes, including pannexin 1 (PANX1) and FERM domain containing 8 (FRMD8), in multiple tumor tissues compared with corresponding normal tissues. The cytokine-related prognostic signature captures key inflammatory and immune-related programs underlying tumor aggressiveness and provides a robust tool for pan-cancer risk stratification with potential clinical utility.

## 1. Introduction

Cytokines are central mediators of intercellular communication within the tumor microenvironment and play critical roles in regulating inflammation, immune responses, tissue remodeling, and cancer progression [1,2,3]. Dysregulated cytokine signaling has been widely implicated in tumor initiation, malignant transformation, immune evasion, and therapeutic resistance across diverse cancer types [1,4,5,6]. Rather than acting as isolated factors, cytokines function through complex and dynamic networks that integrate signals from tumor cells, immune cells, stromal components, and the extracellular matrix [7]. This intricate cytokine landscape contributes to substantial intertumoral and intratumoral heterogeneity, posing major challenges for accurate prognosis assessment and precision oncology [8,9].

Accumulating evidence suggests that cytokine-driven transcriptional programs are closely associated with aggressive tumor phenotypes, including enhanced proliferation, epithelial–mesenchymal transition, metastatic potential, and remodeling of the immune microenvironment [10,11,12]. Specific cytokines such as IL6, IL10, and IL1 family members have been linked to poor clinical outcomes in multiple malignancies, while others may exert context-dependent or even protective effects [13,14]. However, most prior studies have focused on individual cytokines or limited signaling axes, which may fail to capture the coordinated and system-level impact of cytokine networks on cancer biology. A comprehensive evaluation of cytokine-related gene expression patterns may therefore provide more robust and biologically meaningful prognostic information than single-gene biomarkers [15,16,17].

With the increasing availability of large-scale transcriptomic datasets, integrative computational approaches enable systematic dissection of cytokine-related signatures across cancer types. Regularized regression models, such as Least Absolute Shrinkage and Selection Operator Cox regression, allow for the construction of parsimonious prognostic signatures while minimizing overfitting and multicollinearity [18,19,20]. When combined with pan-cancer analyses and independent validation cohorts, these strategies facilitate the identification of conserved cytokine-associated risk patterns and their relevance to patient survival. Moreover, linking cytokine-related signatures to hallmark malignant processes, including cell cycle progression, EMT, angiogenesis, and metabolic reprogramming, may provide mechanistic insights into how inflammatory signaling shapes tumor behavior [21,22,23].

In this study, we systematically investigated cytokine-related genes across pan-cancer cohorts to develop and validate a robust prognostic risk score. By integrating transcriptomic data with survival outcomes, we constructed a cytokine-related gene signature that stratifies patients into distinct risk groups with significantly different clinical prognoses. We further evaluated the distribution and prognostic performance of this signature across cancer types, assessed its clinical utility through nomogram modeling, and explored its biological relevance by correlating the risk score with malignant phenotypes and functional enrichment patterns. Together, our findings provide a comprehensive framework for understanding the prognostic and biological significance of cytokine dysregulation in cancer and highlight its potential value for pan-cancer risk stratification and personalized clinical management.

## 2. Results

### 2.1. Identification and Construction of a Cytokine-Related Prognostic Signature Using LASSO Cox Regression

**Figure 1:**
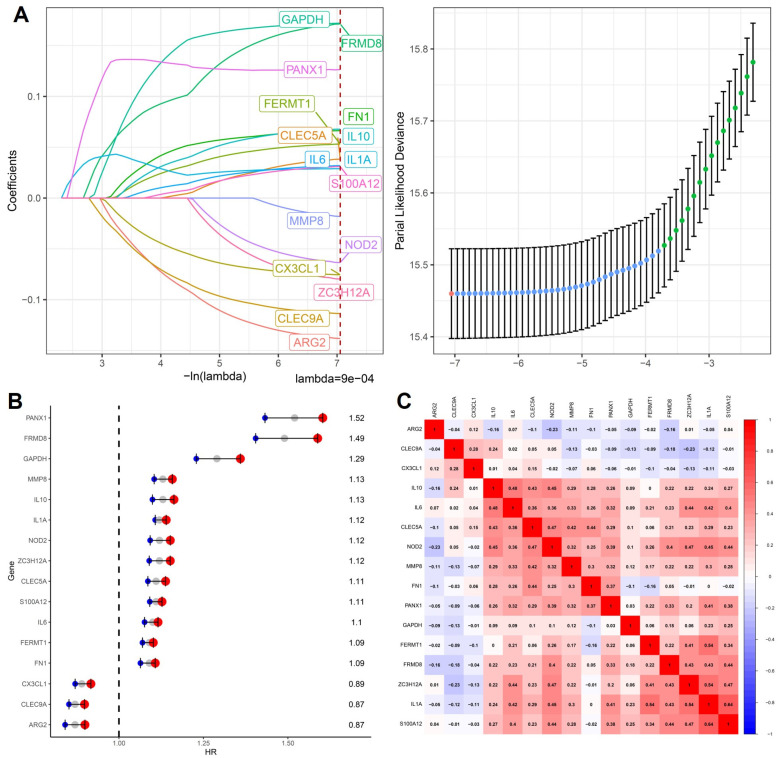
Construction of a cytokine-related prognostic signature. (**A**) LASSO Cox regression coefficient trajectories of cytokine-related genes with the optimal lambda selected by the one-standard-error criterion. The red dashed line indicates the selected optimal lambda value. (**B**) Forest plot shows hazard ratios and 95% confidence intervals of the genes included in the final cytokine-related signature. (**C**) Pearson correlation heatmap of the signature genes, illustrating their expression correlations. with color intensity representing the strength and direction of correlation.

To systematically characterize the prognostic relevance of cytokine-related genes across cancers, we applied LASSO Cox proportional hazards regression to the training cohort to construct a stable and interpretable prognostic signature. As illustrated in Figure 1A, coefficient trajectories demonstrated progressive shrinkage with increasing penalization, indicating effective control of multicollinearity among cytokine-associated candidates.

Using the one-standard-error criterion, an optimal lambda value of 9 × 10^−4^ was selected, resulting in a parsimonious model comprising 16 cytokine-related genes with non-zero coefficients, including PANX1, FRMD8, GAPDH, MMP8, IL10, IL1A, NOD2, ZC3H12A, CLEC5A, S100A12, IL6, FERMT1, FN1, CX3CL1, CLEC9A, and ARG2. These genes collectively represent multiple facets of cytokine-driven tumor biology, encompassing pro-inflammatory signaling, innate immune sensing, leukocyte recruitment, extracellular matrix remodeling, and metabolic adaptation. Subsequent univariate Cox regression analysis revealed marked heterogeneity in prognostic effects among individual genes (Figure 1B), with PANX1, FRMD8, GAPDH, and MMP8 showing hazard ratios greater than 1, consistent with their reported roles in sustaining inflammatory microenvironments and facilitating tumor invasion, whereas ARG2, CLEC9A, and CX3CL1 exhibited protective associations, suggesting potential involvement in immune surveillance or anti-tumor immune modulation. Importantly, the coexistence of both risk-associated and protective cytokine-related genes within the same signature underscores the complex, context-dependent nature of cytokine signaling in cancer progression. To further evaluate redundancy and coordinated regulation within the model, pairwise Pearson correlation analysis was performed.

As shown in Figure 1C, moderate positive correlations were observed among subsets of inflammatory mediators such as IL6, IL10, CLEC5A, and NOD2, reflecting shared activation of innate immune and cytokine signaling pathways, while overall correlation coefficients remained below levels indicative of problematic collinearity, supporting the robustness and stability of the model. Collectively, these results demonstrate that the 16-gene cytokine-related signature integrates diverse yet complementary inflammatory and immune-regulatory signals, providing a biologically coherent framework for downstream prognostic stratification and functional interpretation in pan-cancer analyses.

### 2.2. Pan-Cancer Distribution and Prognostic Relevance of the Cytokine-Related Risk Score

**Figure 2:**
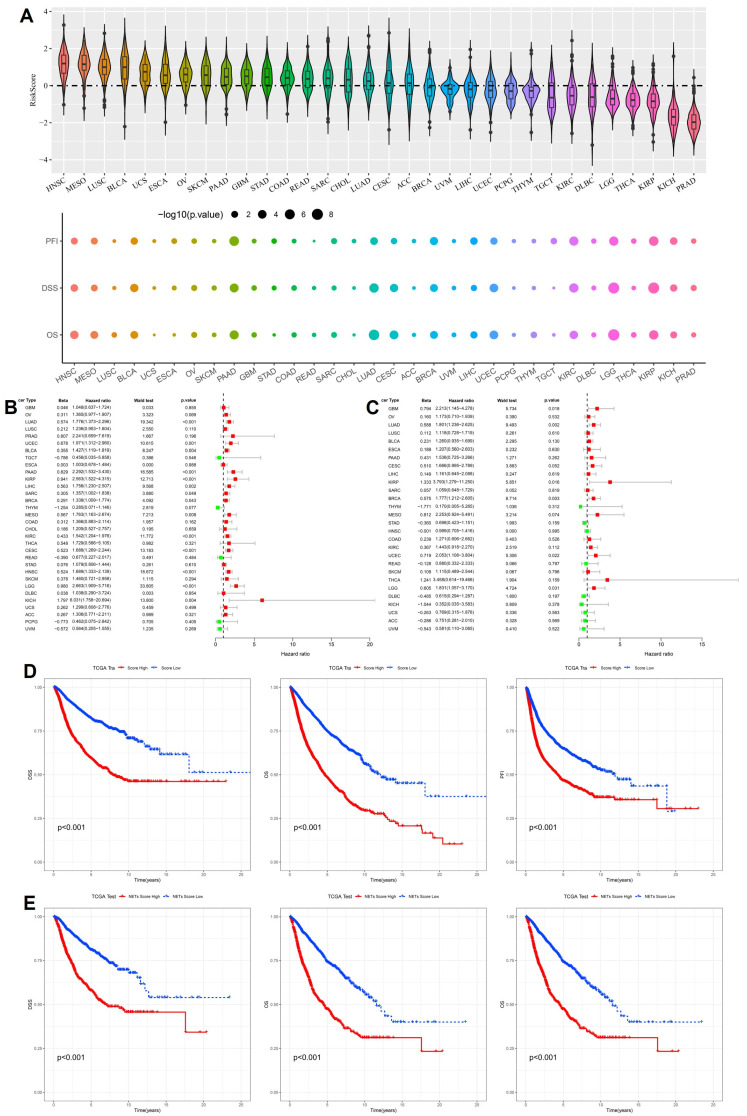
Pan-cancer prognostic performance of the cytokine-related risk score. (**A**) Distribution of cytokine-related risk scores across cancer types and their associations with PFI, DSS, and OS. (**B**) Forest plot shows hazard ratios of the risk score for OS across cancer types in the TCGA training cohort. (**C**) Forest plot shows hazard ratios of the risk score for OS across cancer types in the TCGA test cohort. (**D**) Kaplan–Meier curves for DSS, OS, and PFI in the TCGA training cohort stratified by cytokine-related risk score. (**E**) Kaplan–Meier validation of prognostic stratification in the TCGA test cohort.

Following construction of the 16-gene cytokine-related prognostic signature, we systematically investigated the distribution of the cytokine-related risk score across cancer types and its prognostic implications at the pan-cancer level. As shown in Figure 2A, the risk score exhibited pronounced heterogeneity among different tumor entities. Cancers characterized by chronic inflammation and immune activation, including head and neck squamous cell carcinoma, lung squamous cell carcinoma, bladder cancer, and multiple gastrointestinal malignancies, generally displayed higher risk score distributions, whereas endocrine-related and relatively indolent tumors, such as thyroid carcinoma and prostate adenocarcinoma, showed lower scores. This pattern suggests that cytokine-driven transcriptional programs are strongly shaped by tissue context and tumor-specific immune microenvironments. To further evaluate the prognostic impact of the cytokine-related risk score, univariate Cox regression analyses were conducted across cancer types for progression-free interval (PFI), disease-specific survival (DSS), and overall survival (OS). As illustrated by the bubble plots in Figure 2A, elevated risk scores were significantly associated with adverse survival outcomes in a wide spectrum of malignancies, with bubble size reflecting statistical significance and color intensity indicating effect magnitude. In the TCGA training cohort, forest plot analysis demonstrated that the cytokine-related risk score functioned predominantly as a hazard factor for survival in most cancer types (Figure 2B), with hazard ratios exceeding 1 for OS in multiple tumors, supporting its unfavorable prognostic role. Importantly, these findings were independently validated in the TCGA test cohort, where the majority of cancer types again showed consistent hazard effects associated with higher risk scores (Figure 2C), confirming the robustness and generalizability of the prognostic signal. To assess the clinical discriminative ability of the signature at the cohort level, patients in the TCGA training cohort were stratified into high-risk and low-risk groups according to the median cytokine-related risk score. Kaplan–Meier survival analyses revealed that high-risk patients experienced significantly worse DSS, OS, and PFI compared with low-risk patients (*p* < 0.001, Figure 2D).

This prognostic stratification was reproducibly observed in the TCGA test cohort, where high-risk patients consistently exhibited inferior survival outcomes across all endpoints (Figure 2E). Collectively, these results demonstrate that the cytokine-related risk score captures clinically relevant inflammatory and immune dysregulation signals and provides stable prognostic stratification across cancer types and independent cohorts, underscoring its potential value as a pan-cancer prognostic biomarker.

### 2.3. Cancer-Specific Prognostic Performance of the Cytokine-Related Risk Score in Representative Tumor Types

**Figure 3:**
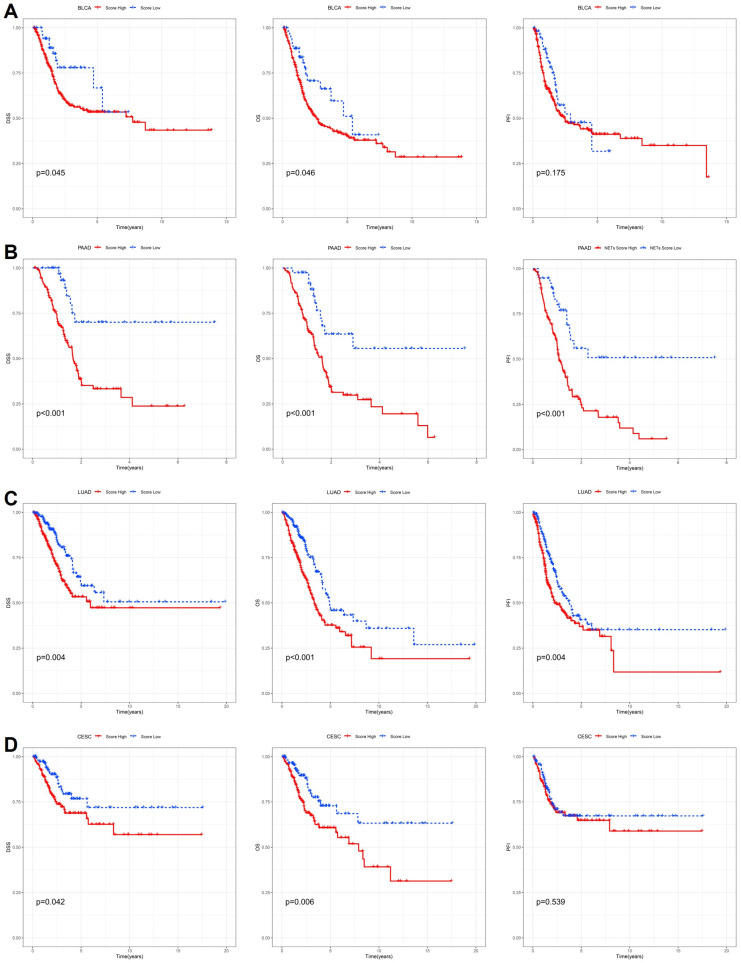
Cancer-specific survival analyses based on the cytokine-related risk score. Kaplan–Meier curves comparing high-risk and low-risk groups stratified by the cytokine-related risk score in (**A**) BLCA, (**B**) PAAD, (**C**) LUAD, and (**D**) CESC. Disease-specific survival (DSS), overall survival (OS), and progression-free interval (PFI) are shown for each cancer type, with *p* values determined by the log-rank test.

To further delineate the cancer-type-specific prognostic value of the cytokine-related risk score, Kaplan–Meier survival analyses were performed in multiple representative malignancies using disease-specific survival (DSS), overall survival (OS), and progression-free interval (PFI) as clinical endpoints. As shown in Figure 3A, patients with bladder urothelial carcinoma (BLCA) stratified into the high-risk group exhibited significantly poorer DSS and OS compared with those in the low-risk group, whereas the difference in PFI did not reach statistical significance. These findings suggest that cytokine-driven transcriptional programs in BLCA are more closely linked to mortality risk rather than early disease progression. In pancreatic adenocarcinoma (PAAD), a highly aggressive and inflammation-associated malignancy, the cytokine-related risk score demonstrated strong and consistent prognostic power across all three endpoints (Figure 3B). High-risk patients showed markedly reduced DSS, OS, and PFI (*p* < 0.001), highlighting the critical role of dysregulated cytokine signaling in driving rapid disease progression and poor outcomes in PAAD. In lung adenocarcinoma (LUAD), elevated risk scores were also associated with significantly worse DSS, OS, and PFI (Figure 3C), indicating that cytokine-related inflammatory and immune-regulatory pathways contribute to both survival and disease progression in this tumor type. By contrast, in cervical squamous cell carcinoma and endocervical adenocarcinoma (CESC), the prognostic impact of the cytokine-related risk score appeared endpoint-dependent (Figure 3D).

While high-risk patients experienced significantly inferior DSS and OS, no significant difference was observed for PFI, suggesting that cytokine-related dysregulation in CESC may preferentially influence long-term survival rather than short-term progression dynamics. Collectively, these cancer-specific analyses demonstrate that although the cytokine-related risk score consistently stratifies patient survival in multiple tumor types, its association with disease progression varies across cancers, reflecting underlying biological heterogeneity and context-dependent roles of cytokine signaling within distinct tumor microenvironments.

### 2.4. Development and Validation of a Cytokine-Related Nomogram for Individualized Survival Prediction

**Figure 4:**
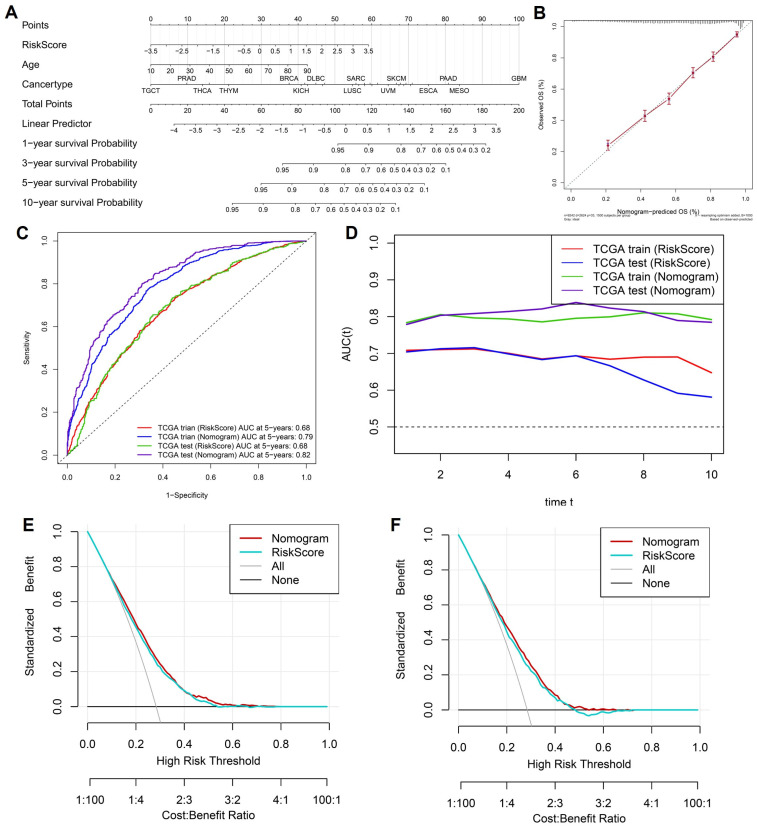
Construction and evaluation of a cytokine-related prognostic nomogram. (**A**) Nomogram integrating the cytokine-related risk score, age, and cancer type to predict 1-, 3-, 5-, and 10-year overall survival. (**B**) Calibration curve comparing nomogram-predicted and observed overall survival probabilities; the dashed line represents the ideal reference line. (**C**) Time-dependent ROC curves at 5 years for the risk score and nomogram in the TCGA training and test cohorts. (**D**) Dynamic AUC profiles over time comparing predictive performance of the risk score and nomogram; the horizontal dashed line indicates the reference AUC value. (**E**,**F**) Decision curve analyses illustrating the standardized net benefit of the nomogram and risk score in the TCGA training and test cohorts, respectively; red and blue lines represent different models, while black and gray lines indicate default strategies.

To enhance the clinical applicability of the cytokine-related risk score and provide an intuitive tool for individualized survival prediction, we integrated the risk score with key clinical variables to construct a comprehensive prognostic nomogram. As shown in Figure 4A, the nomogram incorporates the cytokine-related risk score, patient age, and cancer type, with each variable assigned a weighted point value proportional to its contribution to overall survival risk. By summing the total points for an individual patient, the nomogram enables direct estimation of 1-, 3-, 5-, and 10-year overall survival probabilities, thereby facilitating personalized risk assessment in routine clinical settings. Calibration analysis demonstrated a high degree of concordance between nomogram-predicted and observed survival outcomes. As illustrated in Figure 4B, the calibration curve for overall survival showed close alignment with the 45-degree reference line, indicating that the nomogram provided accurate survival probability estimates with minimal systematic bias. To further quantify predictive performance, time-dependent receiver operating characteristic analyses were performed. The ROC curves at 5 years (Figure 4C) revealed that the nomogram consistently outperformed the cytokine-related risk score alone in both the TCGA training and test cohorts, achieving higher area under the curve values, thereby confirming the added prognostic value of integrating clinical factors with molecular information. This advantage was maintained across time, as demonstrated by the dynamic AUC profiles in Figure 4D, where the nomogram exhibited superior and more stable discrimination ability over long-term follow-up compared with the risk score alone. Beyond statistical performance, the potential clinical utility of the nomogram was evaluated using decision curve analysis.

As shown in Figure 4E,F, the nomogram yielded a higher standardized net benefit than the cytokine-related risk score across a wide range of threshold probabilities in both the training and test cohorts. Importantly, the nomogram consistently outperformed default strategies of treating all or no patients, indicating that its application could improve clinical decision-making by optimizing the balance between benefit and harm. Collectively, these results demonstrate that the cytokine-related nomogram provides accurate, robust, and clinically meaningful survival prediction, supporting its potential role as a practical decision-support tool for risk stratification and personalized management across diverse cancer types.

### 2.5. Association Between the Cytokine-Related Risk Score and Malignant Biological Features Across Pan-Cancer

**Figure 5:**
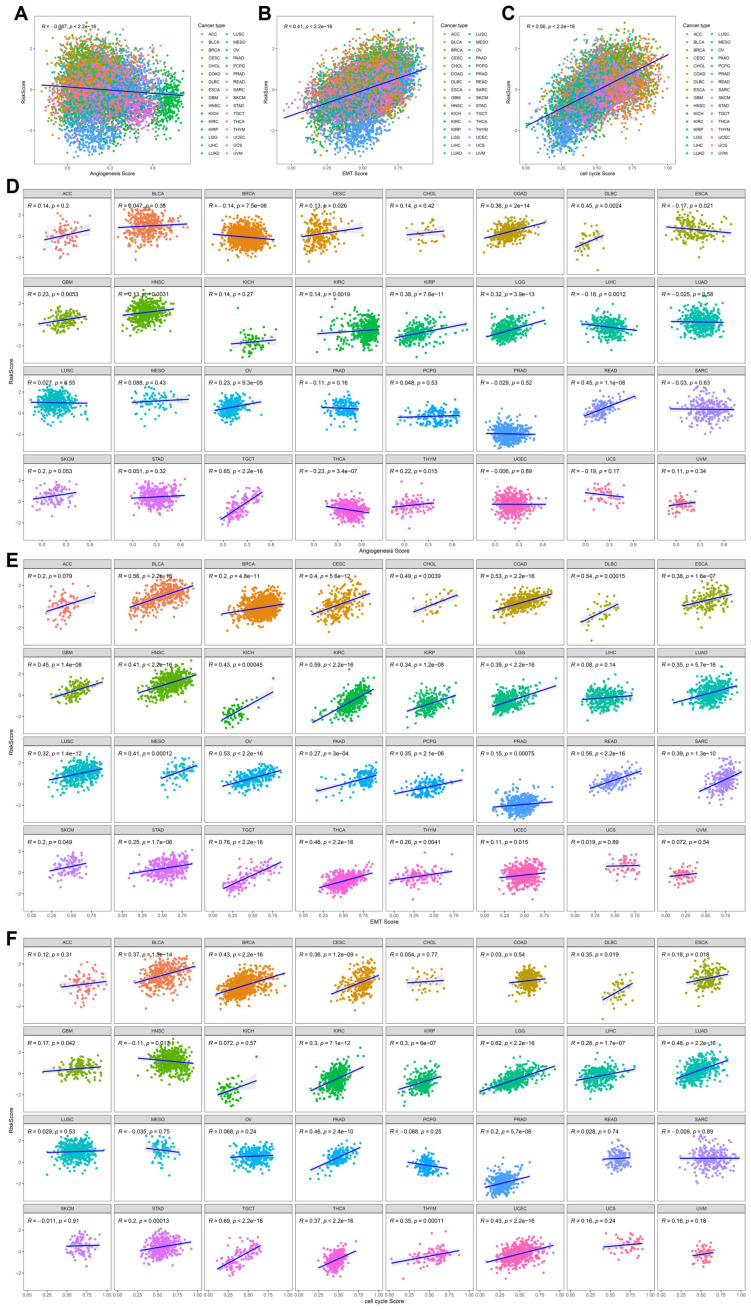
Correlation between the cytokine-related risk score and malignant biological features. (**A**–**C**) Scatter plots showing pan-cancer correlations between the cytokine-related risk score and angiogenesis score, EMT score, and cell cycle score, respectively. (**D**–**F**) Cancer-type-specific correlations between the risk score and angiogenesis score (**D**), EMT score (**E**), and cell cycle score (**F**). Pearson correlation coefficients and corresponding *p* values are indicated in each panel. The blue line represents the fitted regression line, and the gray shaded area indicates the confidence interval.

To explore the biological implications underlying the cytokine-related risk score, we systematically examined its relationship with key malignant features, including angiogenesis, epithelial–mesenchymal transition (EMT), and cell cycle activity, using pathway-level scores across the pan-cancer cohort. As shown in Figure 5A–C, at the global pan-cancer level, the cytokine-related risk score exhibited a significant positive correlation with EMT score and cell cycle score, indicating that tumors with higher cytokine-related risk scores tend to display enhanced mesenchymal characteristics and proliferative capacity. In contrast, a weak but statistically significant negative correlation was observed between the risk score and angiogenesis score, suggesting that cytokine-related signature does not uniformly reflect angiogenic activity and may capture alternative biological programs.

To further delineate tumor-specific patterns, stratified correlation analyses were performed across individual cancer types. As illustrated in Figure 5D, the relationship between the cytokine-related risk score and angiogenesis score varied markedly across cancers. Positive correlations were observed in selected tumor types such as COAD, DLBC, and READ, whereas weak or negative correlations predominated in other cancers, highlighting substantial inter-tumoral heterogeneity. In contrast, the association between the risk score and EMT score was consistently positive across the majority of cancer types (Figure 5E), including BLCA, BRCA, COAD, KIRC, LGG, LUAD, and STAD. Similarly, correlation analyses between the risk score and cell cycle score revealed widespread positive associations across multiple malignancies (Figure 5F), particularly in highly proliferative tumors such as LGG, LUAD, PRAD, and THCA.

Collectively, these findings demonstrate that the cytokine-related risk score is associated with malignant phenotypic features, particularly EMT activation and proliferative signaling, while its angiogenesis appears to be context-dependent. These observations reflect statistically significant associations and should be interpreted as supportive biological correlations rather than direct mechanistic evidence.

### 2.6. Functional Characterization of Cytokine-Related Risk Groups Reveals Distinct Biological Programs

**Figure 6:**
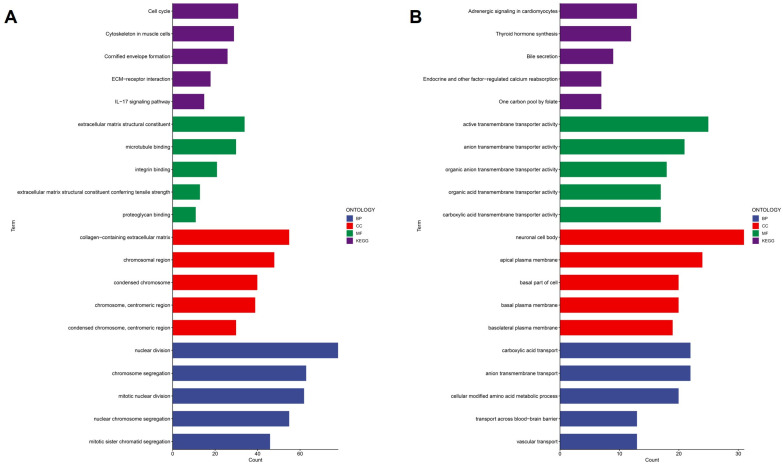
Functional enrichment analysis of cytokine-related risk groups. (**A**) GO and KEGG enrichment results for genes upregulated in the high-risk group. (**B**) GO and KEGG enrichment results for genes upregulated in the low-risk group.

To further characterize the biological patterns associated with the prognostic stratification defined by the cytokine-related risk score, we performed differential expression analysis between high-risk and low-risk groups followed by functional enrichment analyses. Gene Ontology and KEGG pathway enrichment results showed that genes more highly expressed in the high-risk group were associated with processes commonly linked to tumor progression (Figure 6A). At the biological process level, high-risk tumors were enrichment for cell cycle-related terms, including nuclear division, mitotic nuclear division, chromosome segregation, and sister chromatid segregation, consistent with increased proliferative activity. Cellular component analysis highlighted enrichment in chromosomal regions and condensed chromosomes, reflecting active mitosis processes. Molecular function terms were related to extracellular matrix structural constituents, integrin binding, proteoglycan binding, and microtubule binding, suggesting associations with extracellular matrix organization and cytoskeletal dynamics. KEGG pathway analysis identified enrichment of ECM–receptor interaction and IL-17 signaling pathway, indicating involvement proliferative and inflammatory-associated transcriptional programs in the high-risk group. In contrast, genes more highly expressed in the low-risk group exhibited distinct functional profile (Figure 6B). Biological processes were enriched for transmembrane transport activities, including anion and carboxylic acid transport, vascular transport, and transport across the blood–brain barrier. Cellular component terms were dominated by plasma membrane-associated structures, such as apical, basal, and basolateral plasma membranes, as well as neuronal cell bodies. Molecular function enrichment further emphasized organic anion transmembrane transporter activity. KEGG pathways enriched in the low-risk group included adrenergic signaling in cardiomyocytes, thyroid hormone synthesis, bile secretion, and one-carbon pool by folate, reflecting metabolic, and tissue-specific regulatory pathways.

Collectively, these results indicate that high cytokine-related risk groups are associated with distinct transcriptional programs. High-risk tumors display gene expression patterns linked to proliferation, extracellular matrix organization, and inflammatory signaling, whereas low-risk tumors show enrichment of transport and metabolic processes. These findings represent correlative functional associations and provide biological context for the observed prognostic differences.

### 2.7. Immunohistochemical Validation of PANX1 and FRMD8 Expression in Normal and Malignant Tissues

**Figure 7:**
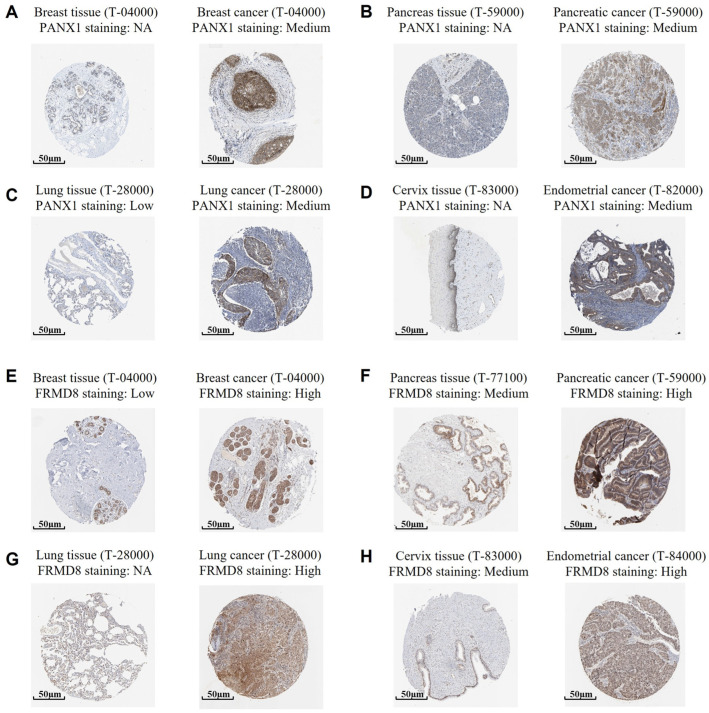
Immunohistochemical validation of PANX1 and FRMD8 expression in normal and cancer tissues. (**A**–**D**) Representative immunohistochemical images showing PANX1 expression in normal tissues and corresponding cancers. (**E**–**H**) Representative immunohistochemical images showing FRMD8 expression in normal tissues and corresponding cancers.

To further support the transcriptomic findings and assess protein-level expression patterns of representative genes within the cytokine-related prognostic signature, immunohistochemical (IHC) staining data were retrieved from the Human Protein Atlas (HPA) database. Representative images from normal and corresponding tumor tissues were examined to qualitatively compare expression patterns across multiple cancer types. As shown in Figure 7A–D, PANX1 protein expression was generally low or undetectable in corresponding normal tissues, including breast, pancreas, lung, and cervix, whereas moderate cytoplasmic staining was observed in matched tumor tissues. Similarly, FRMD8 exhibited low or minimal expression in normal breast and lung tissues, while increased staining intensity was observed in breast cancer, pancreatic cancer, lung cancer, and endometrial cancer specimens (Figure 7E–H). In several tumor types, FRMD8 demonstrated diffuse cytoplasmic expression in malignant tissues compared with limited staining in normal counterparts.

These observations suggest that PANX1 and FRMD8 are higher at the protein level in multiple cancers, consistent with the transcriptomic analyses. However, it should be noted that the HPA database provides representative images rather than quantitative, grade-stratified clinical cohorts. Therefore, these findings serve as supportive qualitative validation of differential expression between normal and tumor tissues rather than definitive evidence of protein-level prognostic association.

Together, the IHC data provide translational support for the inclusion of PANX1 and FRMD8 within the cytokine-related signature while warranting further investigation in well-annotated clinical cohorts to evaluate associations with tumor grade and patient outcomes.

## 3. Discussion

In the present study, we characterized cytokine-related transcriptional programs across pan-cancer cohorts and developed a prognostic signature based on cytokine-associated genes. By integrating large-scale transcriptomic data with survival outcomes, we identified a 16-gene cytokine-related risk score derived from an initial pool of 131 cytokine-related genes curated from GSEA and MSigDB. This signature consistently stratified patients into distinct risk groups with significantly different clinical outcomes across multiple cancer types and independent internal cohorts, supporting the broad prognostic relevance of cytokine-associated transcriptional patterns in cancer. Unlike single-cytokine biomarkers, the composite structure of this signature reflects coordinated and context-dependent inflammatory and immune-related gene expression programs within tumors [24].

The cytokine-related risk score demonstrated heterogeneity across tumor types, likely reflecting differences in tissue-specific inflammatory states and tumor microenvironment composition. Tumors commonly associated with chronic inflammation features tended to exhibit higher risk scores and less favorable outcomes, whereas relatively indolent or hormonally regulated cancers showed lower scores. Importantly, the risk score functioned predominantly as a hazard factor in both training and test cohorts, supporting its reproducibility within the TCGA framework [25]. Cancer-specific analyses further indicated that while the signature consistently stratified survival risk, associations with progression-related endpoints varied across malignancies, suggesting biological context–dependent effects [26]. Beyond prognostic stratification, the cytokine-related risk score was associated with malignant phenotypic features [27]. Elevated risk scores correlated with increased epithelial–mesenchymal transition and cell cycle activity across multiple cancer types, while functional enrichment analyses demonstrated associations with proliferative signaling, chromosome segregation, extracellular matrix organization, and inflammatory pathway activity [28,29]. These findings indicate that the signature captures transcriptional patterns linked to aggressive tumor phenotypes. However, these associations should be interpreted as correlative rather than causal, and they do not establish direct mechanistic regulatory relationships among the included genes or pathways [1,3]. To enhance translation relevance, we integrated the cytokine-related risk score with clinical variables to construct a prognostic nomogram, which demonstrated improved predictive performance compared with the molecular score alone [30]. While this integrated model may support risk stratification, the present study does not establish predictive value for specific treatment modalities. Further validation in therapeutic cohorts will be required to determine whether the signature has utility in guiding treatment decision-making [31].

Nevertheless, several limitations should be acknowledged. First, this study is based primarily on retrospective TCGA transcriptomic data, and external validation in independent datasets or prospective clinical cohorts will be necessary to confirm generalizability [32]. Second, although the signature reflects coordinated cytokine activity, it does not directly capture post-transcriptional regulation or protein-level cytokine dynamics within the tumor microenvironment [33]. Third, while immunohistochemical data from the Human Protein Atlas provided qualitative support for differential protein expression of representative genes, these data were not derived from grade-stratified or outcome-linked clinical cohorts and therefore do not independently establish prognostic protein-level associations. Finally, functional experiments are required to delineate the causal roles of individual genes within the signature [34]. Despite these limitations, our findings provide a pan-cancer framework for evaluating cytokine-related transcriptional patterns in relation to survival and malignant phenotypes. The signature offers a foundation for future mechanistic and clinical investigations aimed at refining cytokine-associated biomarkers in cancer.

## 4. Materials and Methods

### 4.1. Data Collection and Preprocessing

Transcriptomic expression profiles and corresponding clinical annotations were obtained from The Cancer Genome Atlas (TCGA) pan-cancer cohort through the UCSC Xena platform (https://xena.ucsc.edu/ (accessed on 1 December 2025)). Only primary tumor samples with complete survival information were included. Overall survival (OS), disease-specific survival (DSS), and progression-free interval (PFI) were defined according to TCGA standardized clinical endpoints [35]. Gene expression matrices were log2-transformed and subsequently standardized by z-score normalization within each dataset to reduce technical variability. Samples were randomly divided into a training cohort and an internal test cohort at a ratio of 7:3 for model development and validation [36].

### 4.2. Cytokine-Related Gene Curation Based on GSEA and MSigDB

To comprehensively define cytokine-related genes, we systematically curated gene sets associated with cytokine signaling and cytokine-mediated immune responses from Gene Set Enrichment Analysis (GSEA) resources and the Molecular Signatures Database (MSigDB; URL: https://www.gsea-msigdb.org/gsea/msigdb/human/collections.jsp, accessed on 1 December 2025). Specifically, cytokine-related gene sets were retrieved from the MSigDB collections, including Hallmark, C2 curated gene sets, and Gene Ontology (GO) biological process categories related to cytokine activity, cytokine-mediated signaling pathways, inflammatory response, interleukin signaling, and immune regulation [37]. After removing redundant entries and consolidating overlapping genes across gene sets, a total of 131 cytokine-related genes were identified and used as the initial candidate pool for downstream prognostic modeling [38,39].

### 4.3. Survival Analysis and Pan-Cancer Validation

Patients were stratified into high-risk and low-risk groups according to the median cytokine-related risk score within each cohort. Kaplan–Meier survival analyses were performed to compare survival outcomes between groups, and statistical significance was assessed using the log-rank test. Univariate Cox regression analyses were conducted across cancer types to evaluate the prognostic impact of the risk score on overall survival, disease-specific survival, and progression-free interval [24]. The robustness and generalizability of the signature were independently validated in the TCGA test cohort [40].

### 4.4. Nomogram Construction and Performance Evaluation

To improve clinical applicability, a prognostic nomogram (rms package; version 6.8-0 in R; version 4.4.1), was developed by integrating the cytokine-related risk score with key clinical variables, including age and cancer type, using multivariable Cox regression analysis. Calibration curves were generated to assess agreement between predicted and observed survival probabilities [30]. Time-dependent receiver operating characteristic analyses (TimeROC package; version 0.4) were used to evaluate predictive accuracy over time, and dynamic area under the curve values were compared between the risk score alone and the nomogram model. Decision curve analysis was performed to quantify the clinical net benefit of each model across a range of threshold probabilities [41,42].

### 4.5. Functional Enrichment and Pathway Analysis

Differentially expressed genes between high-risk and low-risk groups were identified using predefined statistical thresholds. Gene Ontology (GO) enrichment analyses covering biological processes (BP), cellular components (CC), and molecular function (MF) categories, as well as Kyoto Encyclopedia of Genes and Genomes (KEGG) pathway analyses, were performed using the clusterProfiler package (version 4.10.0) in R [43]. In addition, pathway-level scores for angiogenesis, epithelial–mesenchymal transition, and cell cycle activity were calculated, and Pearson correlation analyses were conducted to assess associations between these malignant features and the cytokine-related risk score at both pan-cancer and cancer-specific levels [44].

### 4.6. Immunohistochemistry (IHC) Analysis

To examine the protein-level expression of representative cytokine-related signature genes, immunohistochemistry (IHC) analyses were performed using publicly available tissue microarray-based staining data (Human Protein Atlas). Representative IHC images for PANX1 and FRMD8 in normal and corresponding tumor tissues were obtained from the HPA (https://www.proteinatlas.org/ (accessed on 1 December 2025), R) [45,46]. Multiple tissue types, including breast, pancreas, lung, cervix, and endometrium, were examined to assess expression patterns across different malignancies. IHC staining intensity was annotated according to the HPA-defined scoring system and categorized as not available (NA), low, medium, or high based on staining strength and the proportion of positively stained cells. Protein expression levels in tumor tissues were qualitatively compared with matched normal tissues to assess differences in staining patterns. Because the HPA database provides representative images rather than quantitative, grade-stratified clinical cohorts, no statistical analysis of tumor grade or outcome-based associations was performed. All IHC images were independently reviewed to ensure consistency of observed staining patterns across tissue types [47].

These analyses were intended to provide qualitative supportive evidence for differential expression patterns consistent with transcriptomic findings, rather than to establish independent protein-level prognostic associations.

### 4.7. Immune Infiltration Analysis

Immune cell infiltration was estimated using single-sample gene set enrichment analysis (ssGSEA) implemented in the GSVA package (version 1.50.0) in R (version 4.4.1; R Foundation for Statistical Computing, Vienna, Austria; https://www.r-project.org/ (accessed on 1 December 2025)). Immune-related gene signatures representing major immune cell populations were used to calculate enrichment scores for each sample.

Pearson correlation analysis was performed to assess the association between immune infiltration scores and the cytokine-related risk score. Data visualization was conducted using the ggplot2 package (version 3.5.1). Default parameters were used unless otherwise specified, and *p* < 0.05 was considered statistically significant.

### 4.8. Statistical Analysis

All statistical analyses were performed using R (4.4.1) software. Two-sided *p* values less than 0.05 were considered statistically significant unless otherwise specified. Continuous variables were summarized using appropriate descriptive statistics, and correlations were evaluated using Pearson correlation coefficients. All analyses followed reproducible research principles.

## 5. Conclusions

In summary, this study systematically delineates the prognostic, biological, and translational significance of cytokine-related gene expression across pan-cancer cohorts. By integrating transcriptomic profiles with survival data, we established and validated a robust 16-gene cytokine-related prognostic signature that consistently stratified patients into distinct risk groups with significantly different clinical outcomes across multiple cancer types and independent cohorts. The cytokine-related risk score was strongly associated with key malignant phenotypes, including enhanced epithelial–mesenchymal transition, cell cycle activation, and extracellular matrix remodeling, providing mechanistic insight into its unfavorable prognostic impact. Importantly, immunohistochemical validation further confirmed the upregulation of representative risk-associated genes, PANX1 and FRMD8, at the protein level in multiple tumor tissues compared with corresponding normal tissues, reinforcing the biological credibility and translational relevance of the signature. Moreover, integration of the cytokine-related risk score with clinical variables into a nomogram improved individualized survival prediction and demonstrated potential clinical utility. Collectively, these findings highlight cytokine-related transcriptional programs as critical determinants of tumor aggressiveness and patient prognosis and support their further development as clinically relevant biomarkers for pan-cancer risk stratification and precision oncology.

## Data Availability

All data involved in this study are available from the corresponding author on request. We employed the Molecular Signatures Database (MSigDB; URL: https://www.gsea-msigdb.org/gsea/msigdb/human/collections.jsp, accessed on 1 December 2025), using the keyword “cytokine” for analysis in this study.

## Abbreviations

The following abbreviations are used in this manuscript:

AUC	area under the curve
BLCA	bladder urothelial carcinoma
CESC	cervical squamous cell carcinoma and endocervical adenocarcinoma
COAD	colon adenocarcinoma
DCA	decision curve analysis
DSS	disease-specific survival
EMT	epithelial–mesenchymal transition
FRMD8	FERM domain containing 8
GSEA	gene set enrichment analysis
IHC	immunohistochemistry
IL	interleukin
KEGG	Kyoto Encyclopedia of Genes and Genomes
KM	Kaplan–Meier
LASSO	least absolute shrinkage and selection operator
LUAD	lung adenocarcinoma
MSigDB	Molecular Signatures Database
OS	overall survival
PAAD	pancreatic adenocarcinoma
PFI	progression-free interval
ROC	receiver operating characteristic
TCGA	The Cancer Genome Atlas
